# A Multi-Scale Model of Disease Transfer in Honey Bee Colonies

**DOI:** 10.3390/insects12080700

**Published:** 2021-08-04

**Authors:** Matthew Betti, Karalyne Shaw

**Affiliations:** 1 Mount Allison University, Sackville, NB E4L 1E2, Canada; 2 Saint Mary’s University, Halifax, NS B3H 3C3, Canada; kshaw@mta.ca

**Keywords:** honey bee, disease transfer, drift, robbing

## Abstract

**Simple Summary:**

Inter-colony disease spread is a impediment to a healthy apiary. A multi-scale mathematical model is built to explore the effects of inter-colony behaviour on the spread of disease. We model different scenarios corresponding to different behaviours exhibited by honey bees. We show that a colony can use certain behaviours to lower the impact of disease on itself, and show that other behaviours are only relevant under specific conditions. The model can be extended to explore an entire apiary or modified to explore the evolutionary underpinnings of these behaviours.

**Abstract:**

Inter-colony disease transfer poses a serious hurdle to successfully managing healthy honeybee colonies. In this study, we build a multi-scale model of two interacting honey bee colonies. The model considers the effects of forager and drone drift, guarding behaviour, and resource robbing of dying colonies on the spread of disease between colonies. Our results show that when drifting is high, disease can spread rapidly between colonies, that guarding behaviour needs to be particularly efficient to be effective, and that for dense apiaries drifting is of greater concern than robbing. We show that while disease can put an individual colony at greater risk, drifting can help less the burden of disease in a colony. We posit some evolutionary questions that come from this study that can be addressed with this model.

## 1. Introduction

Pathogens and disease are now agreed to play an important role in global honeybee colony failure [1]. Honeybee colonies seem to be robust against many individual stressors, but coupled stressors can cause effects greater than the sum of their parts [2]. As honeybees continue to be exposed to stressors such as climate change [3] and sub-lethal pesticide exposure [4], it is important to minimise the risk imposed by disease in order to preserve a colony.

Honeybees are exposed to a host of viral and microbial parasites. The most studied of these are the Varroa mite [5] which can transmit lethal viruses such as deformed wing virus (DWV) [6] and acute paralysis virus (APV) [7] and nosemosis [8] caused by either the spores *Nosema ceranae* or *Nosema apis*. There are other less widely studied viruses and diseases which plague colonies such as cloudy wing virus [9], the newly discovered Lake Sinai virus [10].

Honeybees exhibit behaviours that help combat infection within a colony. Bees will often implement hygienic behaviours [11,12,13] to prevent disease from spreading within a colony. These behaviours consist of removing infected or non-viable brood to stop the spread of infection within a colony.

To prevent disease between colonies, honey bees have a different set of behaviours. One such behaviour is guarding [14], which is when bees stationed at a hive entrance will deny entry to immunocompromised honey bees, bees of another colony, or anything deemed a threat to colony health.

In contrast, some honeybee behaviours facilitate the transfer of infection between colonies. One such behaviour is known as *drift*. Foraging bees can sometimes get confused and return from flight to the wrong hive [15]; if a bee is infected and drifts, they can spread infection to a neighbouring hive [16]. Drifting is worse when hives are visually similar and in close proximity [17]. Honeybees are also known to pillage dying colonies for resources [18]. This is distinct from drift as it is an active behaviour rather than a passive one, and is less dependent on distance given the range of foraging bees, but can still be a transmission route for disease [19].

There are other mechanisms by which disease may pass from one honeybee colony to another. Spillover events between other pollinator species or wild bee populations may cause novel pathogens to infect managed honeybee colonies [20] or vice versa [21] as well as through the environment such as shared foraging patches [20,22].

Diseases in honeybee colonies have been studied mathematically in both the general case [23,24,25,26] and in specific cases of prominent infections [27,28,29]. Recent focus has turned to the study of disease transmission between colonies. A simple model by Muhammad and Eberl illustrates the role of drift using impulsive differential equations [30]. Their results show that drift can allow for the rapid spread of disease through an apiary that drastically reduces the total bee population. In 2019, Bartlett et al. developed a model for networked colonies using an SIR framework to show how colony placement can affect pathogen spread [31].

Infection in honeybee colonies is extremely varied and is confounded by complex social hierarchy and behaviour both within and between hives. This necessitates the use of multi-scale models to aid the understanding of how honeybee behaviour at the individual and colony level effects the transmission of disease. While many diseases have specific, unique routes of transmission, many can be approximated as horizontal transmission between bees. For instance, Varroa mites act as a vector for many viral infections [7,32], but the mite population is dependent on the bee population. As mites often jump from bee to bee [7,33] while feeding, this aspect can be approximated by horizontal transmission. In fact, the mites themselves which weaken bees and lead to health problems [32] can be interpreted as a type of pathogen affecting honey bees. Infections caused by the microsporidian *Nosema* can likewise be approximated by horizontal, bee-to-bee transmission [23], as spores are transmitted through feeding, cleaning, defecating, and transferring pollen and nectar [34]. For this reason, and to increase the tractability of our study, we focus on horizontal transmission between bees on the intra-colony scale.

In the current study, we create a multi-scale model which couples two colonies using the single colony framework introduced in [23], which itself is an extension of the dynamical model of a honey bee colony introduced in [35]. We keep the disease dynamics as general as possible to draw conclusions that can be applied to a wide variety of diseases which affect colonies. The inter-colony behaviours that we will focus on are those pertaining to drift, security, and robbing. We analyse the model using numerical simulations to show the effects of explicit forager and drone drift on the spread of disease as well as under what conditions colony robbing becomes a relevant factor.

## 2. Model

This model extends the model in [23] to incorporate a multi-hive system and inter-colony interactions. In this model, we model two colonies in order to highlight how various behaviours change disease spread between colonies.

The two colonies will be labelled colony 1 and colony 2. Each hive is made up of three classes of bees: the hive bees, *H*, the foragers, *F* and the drones *D*. We ignore brood dynamics in this model, as the goal is to explore a generalised, mass-action infection at the inter-colony level, which is mostly driven by adult bees. The general hive dynamics are adapted from the works in [35,36], and the generalised intra-colony infection is adapted from in [23].

We assume that hive bees (female bees whose duties are contained entirely within the hive) are contained within the hive and are safeguarded against death (in other words, healthy bees are far more likely to become foragers than to die of natural causes). The dynamics of healthy (i.e., susceptible) hive bees are governed by the equation

(1) $$\begin{array}{cc} \frac{d{}_{1}H{}_{S}}{dt} & =p_{1}LS_{1}\left({}_{1}H,{}_{1}f\right)-R_{1}\left({}_{1}H,{}_{1}F\right){}_{1}H{}_{S}-{}_{1}H{}_{S}\Upsilon_{H}\left({}_{1}H{}_{I},{}_{1}F{}_{I},{}_{1}D{}_{I}\right) \end{array}$$

(2) $$\begin{array}{cc} \frac{d{}_{2}H{}_{S}}{dt} & =p_{1}LS_{2}\left({}_{2}H,{}_{1}f\right)-R_{2}\left({}_{2}H,{}_{1}F\right){}_{2}H{}_{S}-{}_{2}H{}_{S}\Upsilon_{H}\left({}_{1}H{}_{I},{}_{1}F{}_{I},{}_{1}D{}_{I}\right) \end{array}$$

where the subscripts 1 or 2 determine which colony the bees are from, *S* stands for susceptible bees, and *I* indicates infected bees. The parameter $p_{1}$ is the proportion of eggs laid by the queen of colony 1 which are fertilised (and thus result in female bees), and *L* is the number of eggs laid per day on average by the queen.

The function $\Upsilon_{H}\left({}_{1}H{}_{I},{}_{1}F{}_{I},{}_{1}D{}_{I}\right)$ is an infection transmission function between infected bees and susceptible bees. It is given as

(3) $$\begin{array}{c} \Upsilon_{H}\left({}_{1}H{}_{I},{}_{1}F{}_{I},{}_{1}D{}_{I}\right)=\left(\beta_{HH}{}_{1}H{}_{I}+\beta_{HF}\left({}_{1}F{}_{I}+{}_{2}^{1}F{}_{I}\right)+\beta_{HD}{}_{1}D{}_{I}\right) \end{array}$$

where $\beta_{HH}$ is the interaction term between hive bees, $\beta_{HF}$ is the interactions between foragers and hive bees, and $\beta_{DH}$ is the interactions between drones and hive bees. The subscript on Υ denotes the susceptible population being interacted with. Therefore, there are other interactions not shown here, namely, $\beta_{DF}$, $\beta_{DD}$, and $\beta_{FF}$. In practice, we take an average β value and the term reduces to

(4) $$\begin{array}{c} \Upsilon \left({}_{1}H{}_{I},{}_{1}F{}_{I},{}_{1}D{}_{I}\right)=\beta \left({}_{1}H{}_{I}+{}_{1}F{}_{I}+{}_{2}^{1}F{}_{I}+{}_{1}D{}_{I}\right) \end{array}$$

These two governing equations, Equations (1) and (2), do not interact with each other as we assume hive bees of one colony are not mixing with other colonies directly.

The two functions S(H,f) and R(H,F) are survival of brood and recruitment to foraging, respectively, where ${}_{1}H=\left({}_{1}H_{S},{}_{1}H{}_{I}\right)$ and ${}_{1}F=\left({}_{1}F_{S},{}_{1}F{}_{I}\right)$ and similarly for colony 2. These terms were first introduced in [35,36]. We have adapted and modified these terms to the following:

(5) $$\begin{array}{c} S_{1}\left(H,f\right)=\left(\frac{H_{S}+H_{I}}{k_{1}+H_{S}+H_{I}}\right)\left(\frac{f}{b_{1}+f}\right) \end{array}$$

and

(6) $$\begin{array}{c} R_{1}\left(H,F\right)=\alpha_{1}\left(1-\sigma_{1}\frac{F_{S}+F_{I}}{H_{S}+H_{I}+F_{S}+F_{I}}\right). \end{array}$$

The parameters $k_{1}$ and $b_{1}$ are the amount of hive bees and food required to ensure 50% survival of the brood, parameter $\alpha_{1}$ is the rate of recruitment to foraging in the absence of foragers, and $1\;/\;\sigma_{1}$ is the optimal proportion of foragers in a colony. We have similar equations for colony 2.

Infected hive bees are created when an infected bee interacts with a susceptible hive bee. These infected hive bees are then susceptible to infection-related death at rate $d_{H}$.

(7) $$\begin{array}{cc} \frac{d{}_{1}H{}_{I}}{dt} & ={}_{1}H{}_{S}\Upsilon_{H}\left({}_{1}H{}_{I},{}_{1}F{}_{I}\right)-R_{1}\left({}_{1}H,{}_{1}F\right){}_{1}H{}_{I}-d_{H}{}_{1}H{}_{I} \end{array}$$

(8) $$\begin{array}{cc} \frac{d{}_{2}H{}_{I}}{dt} & ={}_{2}H{}_{S}\Upsilon_{H}\left({}_{2}H{}_{I},{}_{2}F{}_{I}\right)-R_{2}\left({}_{2}H,{}_{2}F\right){}_{2}H{}_{I}-d_{H}{}_{2}H{}_{I} \end{array}$$

Healthy (i.e., susceptible) foragers, $F_{S}$, are created through recruitment of hive bees and are susceptible to natural death at rate μ, as well we incorporate a drifting term which allows foragers to visit other colonies. We denote these “drifted foragers” with two left indices. For example, ${}_{2}^{1}F{}_{S}$ is a healthy forager from colony 2 who is currently in colony 1. This separation has multiple advantages; it allows for asymmetry in the model (we can allow migrant foragers an increased probability of returning to their proper colony), it allows us to account for their average age before drifting (if we were to add them to ${}_{2}F_{S}$ they would then live on average $\mu_{2}$ days from the day they drifted), and we can track migrant foragers and validate against experimental results. Given this, there are two equations per colony for healthy foragers:

(9) $$\begin{array}{cc} \frac{d{}_{1}F{}_{S}}{dt} & =R\left({}_{1}H,{}_{1}F\right){}_{1}H_{S}-\mu_{1}{}_{1}F{}_{S}-d_{12}{}_{1}F{}_{S}+r_{1}{}_{2}^{1}F{}_{S}-{}_{1}F_{S}\Upsilon \left({}_{1}H{}_{I},{}_{1}F{}_{I}\right) \end{array}$$

(10) $$\begin{array}{cc} \frac{d{}_{1}^{2}F{}_{S}}{dt} & =d_{21}{}_{2}F{}_{S}-r_{2}{}_{1}^{2}F{}_{S}-{}_{1}^{2}F{}_{S}\Upsilon \left({}_{1}H{}_{I},{}_{1}F{}_{I}\right)-\frac{\mu_{1}}{\mu_{1}+r_{1}}{}_{1}^{2}F{}_{S} \end{array}$$

(11) $$\begin{array}{cc} \frac{d{}_{2}F_{S}}{dt} & =R\left({}_{1}H,{}_{1}F\right){}_{2}H_{S}-\mu_{2}{}_{1}F{}_{S}-d_{21}{}_{1}F{}_{S}+r_{2}{}_{1}^{2}F{}_{S}-{}_{2}F_{S}\Upsilon \left({}_{2}H{}_{I},{}_{2}F{}_{I}\right) \end{array}$$

(12) $$\begin{array}{cc} \frac{d{}_{2}^{1}F{}_{S}}{dt} & =d_{21}{}_{2}F{}_{S}-r_{1}{}_{2}^{1}F{}_{S}-{}_{2}^{1}F{}_{S}\Upsilon \left({}_{2}H{}_{I},{}_{2}F{}_{I}\right)-\frac{\mu_{2}}{\mu_{2}+r_{2}}{}_{2}^{1}F{}_{S} \end{array}$$

where $d_{12}$ is the probability of drift from colony 1 to colony 2, $d_{21}$ is the probability of drift from colony 2 to colony 1, and μ is the rate of natural death of foragers.

Infected foragers have similar dynamics to healthy foragers, but there are two routes of creation (infected foragers can be recruited to foraging from infected hive bees, or healthy foragers that become infected). As well, infected foragers will die either through natural death or by death due to infection, $d_{F}$. Equations (13)–(16) show the governing behaviour of infected foragers. The notable changes from the susceptible forager equations are highlighted in blue.

(13) $$\begin{array}{cc} \frac{d{}_{1}F{}_{I}}{dt} & =R\left({}_{1}H,{}_{1}F\right){}_{1}H_{I}-\left(\mu_{1}+d_{F}\right){}_{1}F{}_{S}-d_{12}{}_{1}F{}_{S}+r_{1}{}_{2}^{1}F{}_{S}+{}_{1}F_{S}\Upsilon \left({}_{1}H{}_{I},{}_{1}F{}_{I}\right) \end{array}$$

(14) $$\begin{array}{cc} \frac{d{}_{1}^{2}F{}_{S}}{dt} & =d_{21}{}_{2}F{}_{S}-r_{2}{}_{1}^{2}F{}_{S}+{}_{1}^{2}F{}_{S}\Upsilon \left({}_{1}H{}_{I},{}_{1}F{}_{I}\right)-\frac{\mu_{1}+d}{\mu_{1}+r_{1}+d}{}_{1}^{2}F{}_{S} \end{array}$$

(15) $$\begin{array}{cc} \frac{d{}_{2}F_{I}}{dt} & =R\left({}_{1}H,{}_{1}F\right){}_{2}H_{I}-\left(\mu_{2}+d_{F}\right){}_{1}F{}_{S}-d_{21}{}_{1}F{}_{S}+r_{2}{}_{1}^{2}F{}_{S}+{}_{2}F_{S}\Upsilon \left({}_{2}H{}_{I},{}_{2}F{}_{I}\right) \end{array}$$

(16) $$\begin{array}{cc} \frac{d{}_{2}^{1}F{}_{S}}{dt} & =d_{21}{}_{2}F{}_{S}-r_{1}{}_{2}^{1}F{}_{S}+{}_{2}^{1}F{}_{S}\Upsilon \left({}_{2}H{}_{I},{}_{2}F{}_{I}\right)-\frac{\mu_{2}+d}{\mu_{2}+d+r_{2}}{}_{2}^{1}F{}_{S} \end{array}$$

Drones are also a potential vector of infection between colonies. Drone behaviours are incredibly simple, they are born of unfertilised eggs, and tend to be long-lived. Drones live on average 90 days, but at certain times of the year death can be accelerated through mating in spring/early summer [37], or through the mass eviction of drones in autumn by worker bees [38]. Similar to foragers, drones may drift between hives [19]. Our governing equations for healthy drones in colony 1 are given as

(17) $$\begin{array}{cc} \frac{d{}_{1}D{}_{S}}{dt} & =\left(1-p_{1}\right)LS_{1}\left(H,f\right)-{}_{1}D_{S}\Upsilon \left({}_{1}H{}_{I},{}_{1}F{}_{I}\right)-\mu_{D}{}_{1}D_{S} \end{array}$$

(18) $$\begin{array}{cc} \frac{d{}_{1}^{2}D{}_{S}}{dt} & ={\hat{d}}_{21}{}_{2}D{}_{S}-{\hat{r}}_{2}{}_{1}^{2}D{}_{S}-{}_{1}^{2}D{}_{S}\Upsilon \left({}_{1}H{}_{I},{}_{1}F{}_{I}\right)-\frac{\mu_{D1}}{\mu_{D1}+{\hat{r}}_{2}}{}_{2}^{1}F{}_{S} \end{array}$$

with similar equations for colony 2 and infected equations build in the same way as those for hive bees and foragers.

## 3. Results

The model is run with parameters given in Table 1. For simplicity we set all parameters equal for both colonies, and set $\beta =\beta_{HH}=\beta_{FH}=\beta_{HD}=\beta_{HF}$, $d_{12}=d_{21}=r_{1}=r_{2}$ and ${\hat{d}}_{12}={\hat{d}}_{21}={\hat{r}}_{1}={\hat{r}}_{2}$.

**Figure 1:**
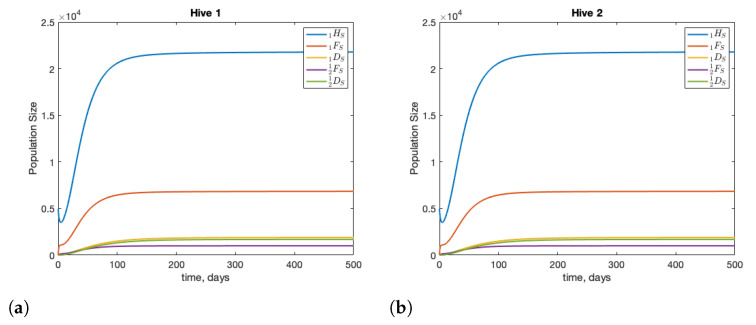
The dynamics of two healthy colonies with interactions through forager and drone drifting for colony 1 (**a**) and colony 2 (**b**). The model is started with initial conditions ${}_{1}H_{S}\left(0\right)={}_{2}H_{S}\left(0\right)$ = 10,000, and all other compartments with 0 individuals. Food is considered abundant and infinite. As identical parameter sets are used for both colonies, the dynamics of both colonies are the same.

**Figure 2:**
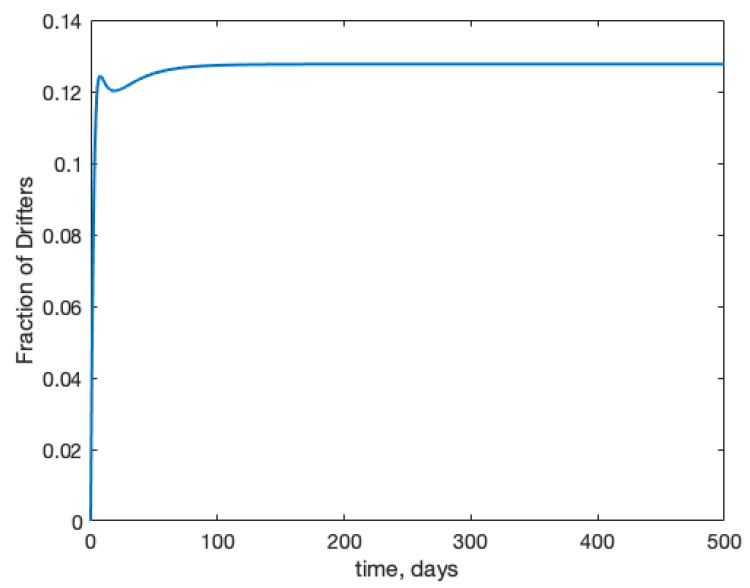
With the drifting rate set at 10%, we see that on average 12% of the foragers in a colony are foreign. These results are consistent with experimental findings.

The healthy dynamics of two colonies are shown in Figure 1. Each colony is started with 10,000 hive bees, no foragers, and no drones. This roughly replicates post-winter conditions. Within 1–2 months, each colony is strong and stable. Without infection, and with two identical colonies, we see that the fraction of drifted foragers in a colony, under the given parameter set is roughly 12%; shown in Figure 2. These results are consistent with biological observations: see in [44] for drift values, and in [45] for colony size.

**Figure 3:**
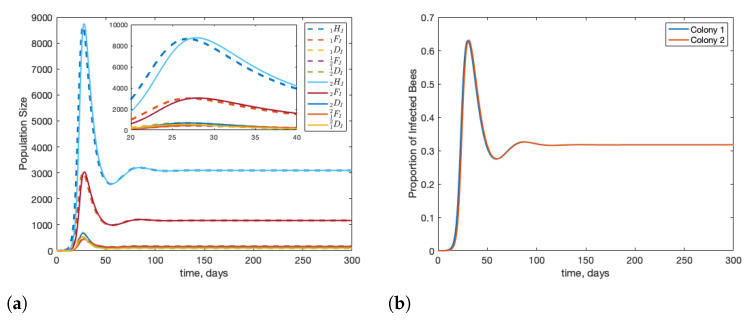
Beginning from equilibrium, we introduce one infected forager into colony 1 (${}_{1}F_{I}\left(0\right)=1$). We see that infection can travel quickly between two colonies with little delay. Note that the infection in the second colony peaks slightly higher than in the source colony. Panel (**a**) shows the population of infected bees over time and panel (**b**) shows the proportion of bees in each colony that are infected over time.

In Figure 3, we first run the model to reach the disease-free equilibrium and then introduce infection. This is to simulate infection being introduced in mid-summer, when colonies are at full strength. In this case, we see that infection travels between colonies quickly, and despite the colonies being identical, the peak infected population in the second colony is slightly higher than in the source colony. In panel (b) of Figure 3, we see the proportion of bees that are infected in each colony. This is important as a proxy for future risk to the colony as the stress of infection can amplify the effects of other stressors.

### 3.1. Scenario: Drone Drift vs. Forager Drift

**Figure 4:**
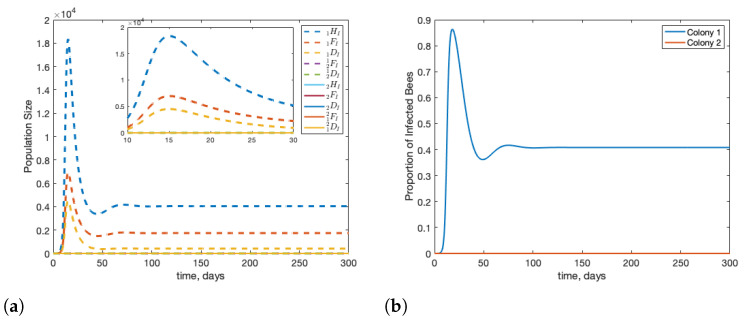
Beginning from equilibrium, we introduce one infected forager into colony 1 (${}_{1}F_{I}\left(0\right)=1$). We see that infection is more severe and poses greater long-term risk when bees are not able to drift between colonies. Subfigure (**a**) shows the infected population in the hives, subfigure (**b**) shows the percentage of infected bees in each colony.

We can modify the model by looking at the impact of forager drift versus drone drift on infections within a two colony system. When compared to Figure 3, Figure 4 shows that drift allows the stress of infection to be spread across both colonies. Figure 4 shows the case when there is no drift, in other words, $d_{12}=d_{21}=r_{1}=r_{2}=0$ and ${\hat{d}}_{12}={\hat{d}}_{21}={\hat{r}}_{1}={\hat{r}}_{2}=0$. We see that when the infection is limited to the source colony, the peak number of infected bees is much higher than when bees are able to drift between colonies. We also see that the proportion of bees infected in colony 1 is higher, by roughly 10%.

**Figure 5:**
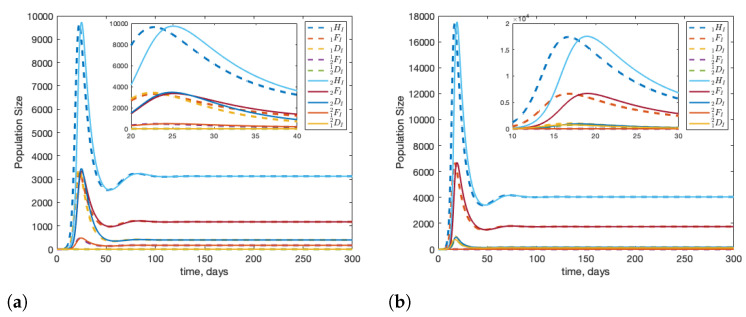

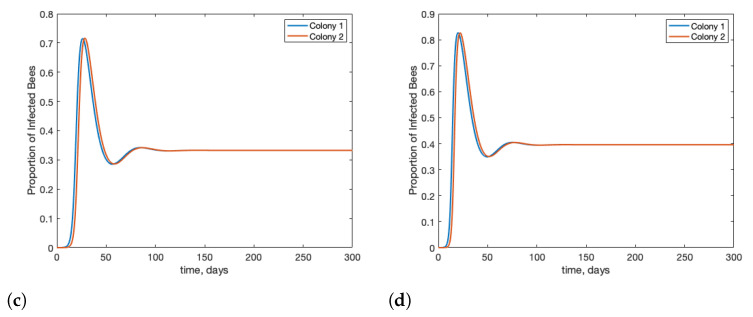
Beginning from equilibrium, we introduce one infected forager into colony 1 (${}_{1}F_{I}\left(0\right)=1$). Subfigure (**a**) shows the impact of only forager drift on the system of two colonies; ${\hat{d}}_{12}={\hat{d}}_{21}={\hat{r}}_{1}={\hat{r}}_{2}=0$ and all other parameters as in Table 1. Subfigure (**b**) shows the impact of only drone drift; $d_{12}=d_{21}=r_{1}=r_{2}=0$ and all other parameters as in Table 1. Comparing panels (**c**,**d**), we see that forager drift is mainly responsible for alleviating infection pressure in the colony as when forager drift is removed, the situation in colony 1 is very similar to when there is no drift at all.

In Figure 5, we show the impact of forager drift (subfigure (a)) in the absence of drone drift and the impact of drone drift (subfigure (b)) in the absence of forager drift. We see that despite their increased rate of drift, if foragers are not allowed to drift, both colonies see an increase in infection, and the infection peaks roughly 10 days earlier than if both drones and foragers are able to drift. We see that thee same qualitative result is true if only foragers are able to drift, but it is much less pronounced quantitatively.

### 3.2. Scenario: Filtering Infected Bees

**Figure 6:**
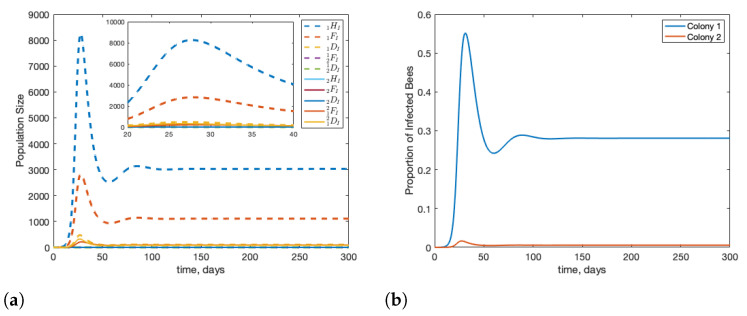

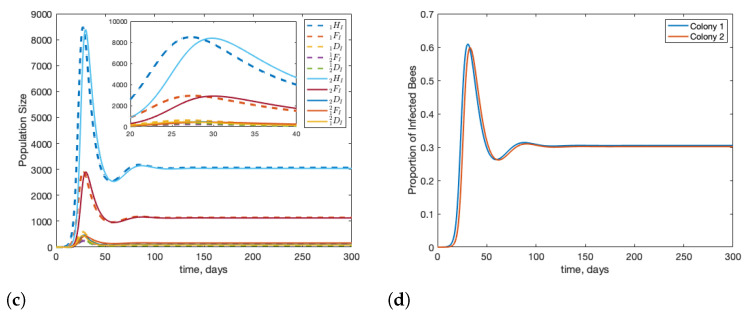
Beginning from equilibrium, we introduce one infected forager into colony 1 (${}_{1}F_{I}\left(0\right)=1$). In this scenario, we allow healthy foragers and drones to drift between colonies, but infected bees are detected and are removed. In panels (**a**,**b**), we assume there is a perfect filter for infected hive bees; in panels (**c**,**d**), we assume this guarding behaviour is 50% effective.

Another behaviour some honey bee colonies can show is to disallow drifting of compromised bees [46]. In Figure 6, we simulate colony 2 discriminating against the drift of infected bees from colony 1, but colony 1 does not discriminate at all. We see that a perfect filter can save colony 2 in much the same way as no drift; as the guarding bees at colony 2 will often kill the trespassing bees [14], we also see a healthier colony 1 because of the behaviour of hive 2. The figure also shows the case where only 50% of infected drifting bees are stopped and we see that this is not enough to prevent infection in colony 2.

### 3.3. Scenario: Robbing Behaviour

**Figure 7:**
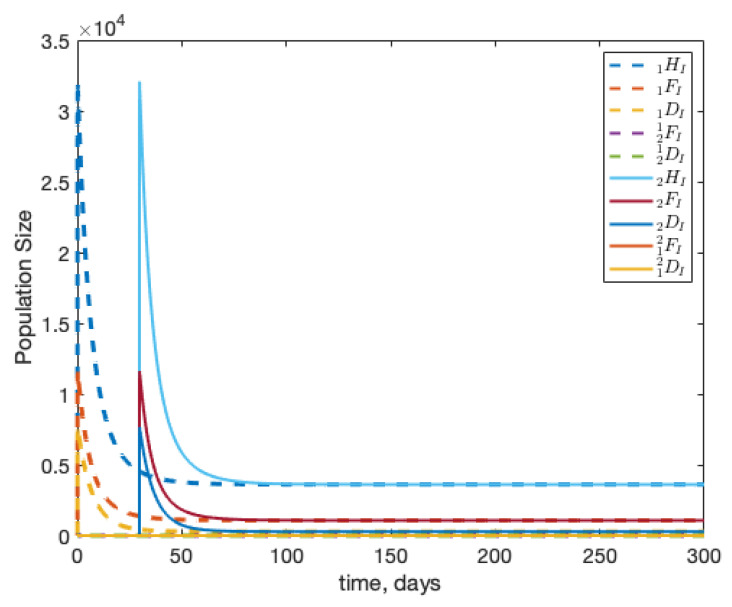
Beginning from equilibrium, we introduce one infected forager into colony 1 (${}_{1}F_{I}\left(0\right)=1$). In this scenario, we remove drift but allow colony 2 to rob colony 1 of resources as the colony begins to die. We see that in this case the colonies behave independently.

Another behaviour that bees show is robbing dying colonies for resources [19]. In this situation, healthy foragers from colony 2 will target a dying colony for its resources, often picking up infection along the with the resources [19]. We simulate this situation by inserting an infected forager into colony 1, and running the simulation until the population of colony 1 is below 6000 bees, we then insert an infected forager into colony 2. This is to simulate robbing from a dying colony. When we do this without any drift, we see in Figure 7 that the the colonies independently as expected. Note for these results we set $\beta =2\times 10^{-3}$ to ensure colony 1 collapses. As mentioned above, ignoring drift can be interpreted as simulating wild colonies that are often far enough apart that drifting is not an issue [17].

**Figure 8:**
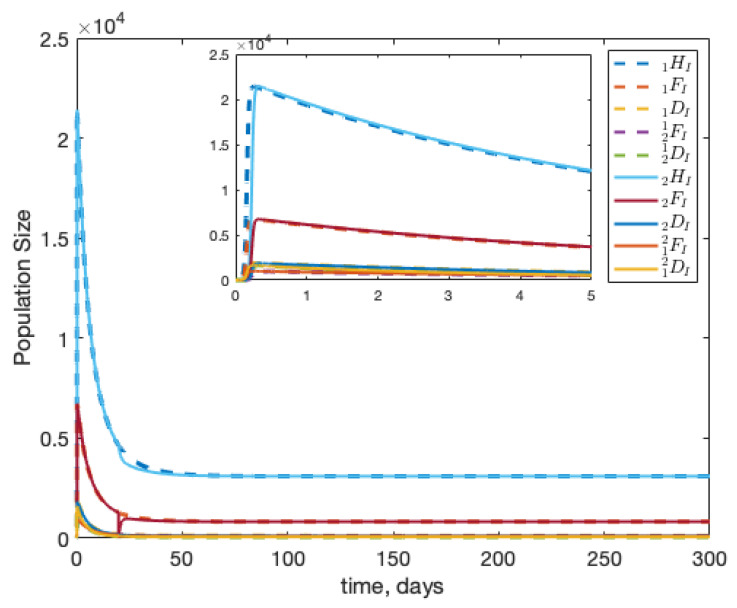
Beginning from equilibrium, we introduce one infected forager into colony 1 (${}_{1}F_{I}\left(0\right)=1$). In this scenario, we allow drifting between colonies and allow colony 2 to rob colony 1 of resources as the colony begins to die. We see that in this case the effects of robbing are washed out.

When we combine the effects of drift and robbing, we see that infection spread by drift dominates. This is visualised in Figure 8.

### 3.4. Basic Reproduction Number

The model is too complex for the use of analytical techniques to determine a basic reproduction number to get a sense of severity of disease. We can however estimate the basic reproduction number from our numerical simulations. Using the heuristic that the proportion of infected individuals, *p*, at the endemic equilibrium is related by

$$\begin{array}{c} p=1-\frac{1}{R_{0}} \end{array}$$

we can show that our two values of β in our study correspond to a reproduction number of $R_{0}\approx 1.67$ when $\beta =5\times 10^{-4}$, and $R_{0}\approx 20$ when $\beta =2\times 10^{-3}$, in a single colony. These values are in the same range as those used in [23,31].

### 3.5. Varying Drift & Reproduction Number

**Figure 9:**
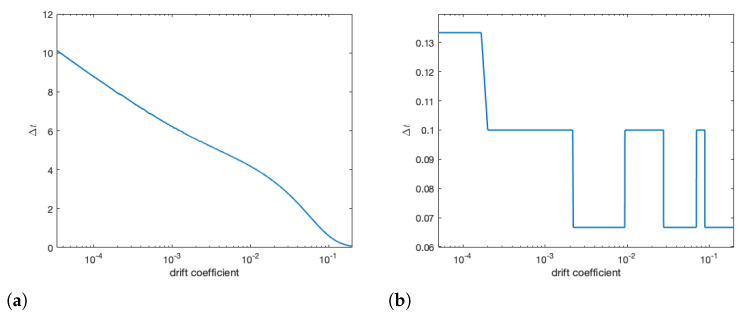
With all other parameters being equal, we change the drift coefficient and measure the time between peak infections in colony 1 and colony 2. We can see for low β (panel (**a**)) that increasing the drift coefficient (i.e., decreasing the distance between colonies) exponentially decreases the time it take an infection to reach colony 2. When β is high (panel (**b**)), we see that there is less importance on drift, it only need be present.

As drift is dependent on distance between the colonies, we examine the effects of varying drift on colony behaviour. In Figure 9, we see that lowering drift by distancing colonies can slow spread of infection between colonies by upwards of 10 days. In panel (a), when $\beta =5\times 10^{-4}$, we see that as drift rate increases (i.e., distance between colonies decreases), there is a rapid decrease in the time it takes infection to spread from one colony to another. We see in panel (b) that when $\beta =2\times 10^{-3}$, the drift coefficient plays little role in spread and drift need only be present.

**Figure 10:**
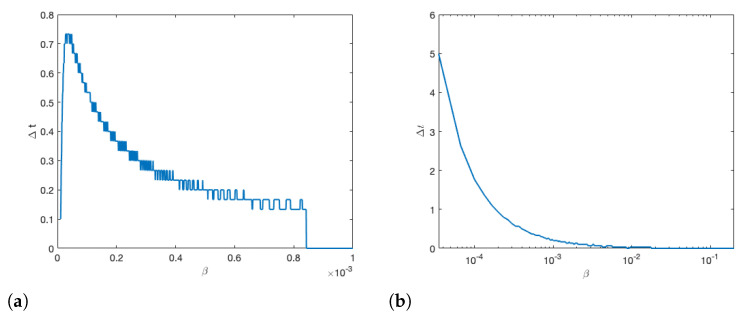
With all other parameters being equal, we change β and measure the time between peak infections in colony 1 and colony 2. We can see that increasing β exhibits exponentially decreases in the time it take an infection to reach colony 2, but the change is quite small. In panel (**a**), we use a drift coefficient of 0.1, and in panel (**b**), we use a drift coefficient of $1\times 10^{-4}$.

In Figure 10, we vary β and measure the time between peak infections in colony 1 and colony 2. We see that increased infectiousness between bees does not have as large an impact on inter-colony transmission as drift. In panel (a) we set the forager drift coefficients to 0.1 and in panel (b) we set the forager drift coefficient to $1\times 10^{-4}$ for comparison so show that the insensitivity to β holds.

## 4. Discussion

In this study, we explore the dynamics of disease between two coupled honey bee colonies. We use a system of two colonies so that we can clearly highlight the effects of infection in one colony on another. We tune the drift parameters so that the number of expatriated bees from each colony is roughly 12% at any given time. This is in line with observation [17], although drift as high as 80% has been observed [47].

When exploring the interplay between drift and disease spread, we specifically choose disease parameters such that $R_{0}$ is low. This allows our results to highlight the qualitative changes induced by our different behavioural scenarios. As we can see in Figure 7 and Figure 8, a larger $R_{0}$ allows an infection to spread incredibly rapidly within a colony. Studying under a lower $R_{0}$ helps highlight the changes induced by changing behaviours.

Primarily, we notice that when bees are able to drift between colonies, the severity of disease in *both* colonies is reduced. In edge cases, with many colonies, this may help colonies survive in the face of infection instead of dying out. We can expect that if we added more colonies to this model, we may see a further reduction in severity. This may help explain why many diseases that affect colonies are sublethal [48,49,50] as the population of colonies “share” the stress of infection.

We use the proportion of infected bees in surviving infected colonies as a proxy for future risk from other points of stress. We posit that colonies with high levels of infection will be more susceptible to failure from other stresses. In this sense, we see that drifting can lower future risk in the meta-population by reducing the infection load in each individual colony. This is a particularly interesting result as it seems counterintuitive. That being said, drifting is not the only avenue by which one can reduce stress within an infected colony. Certain types of infections may be better treated by queen replacement [51], better access to forage, etc. There may be cases when it is beneficial to have two working colonies with 60% of the bees healthy versus one failed colony and one healthy colony (for an average of 50% of the bees in the meta-population).

To contrast the above point, the fact that approximately 30% of bees within a colony are infected put the colony at increased risk from other stressors [2,52] and seasonal effects [23,24,53]. In this sense, from the perspective of the healthy colony, it is beneficial to prevent infection. This could be one avenue by which colonies developed guarding behaviour. Current research suggests that some viruses are adapting to circumvent guarding behaviour [54], which may lead to further evolutionary pressure against drifting, causing honey bees to become more territorial.

Our results also show that robbing is mostly a cause for disease spread between colonies when drifting is rare, and in this scenario, honey bee colonies mostly act independently. With commercial colonies often being identical and kept within close proximity, the model predicts that disease spread through drift occurs faster and more frequently than spread through resource robbing. This suggests that disease mitigation in colonies should first focus on reducing drift, which would allow beekeepers more time to isolate dying colonies before they can be robbed.

Finally, we see that extremely infectious pathogens (high β) or extremely dense colonies will results in rapid transmission between colonies (Figure 9 and Figure 10 regardless of the state of the other parameter.

There are also many ways to extend the single colony model to account for different behaviours as well. By including a queen compartment with its own life cycle, one could study the effects of queen replacement on colony infection. The parameters of the model are also set to reflect commercial beekeeping efforts in Canada/United States using *Apis mellifera*. The model could be reparametrized for hobbyist beekeepers, *Apis cerana*, or displaced/imported bees to a new environment. This is dependent on available data for each of these cases. Some of these studies would be better suited to an agent-based simulation such as those in [33,55].

Moreover, extensions of the multi-scale framework to include other pollinators are also interesting. Combining multiple pollinators in a spatially explicit model may lead to insights into how and when wild pollinators are at greatest risk of disease spillover [56,57,58] from managed honeybee colonies. Such a spatially heterogeneous model will also allow for the study of infections spread through foraging such as in the case of *Nosema* whose spores can be transmitted via bees foraging at the same flower.

From an evolutionary perspective, we see that drifting and closer colonies may have been relevant in reducing overall stress. This could have been beneficial to the meta-population when environmental stresses (i.e., climate change and pesticide use) were less imposing on a colony’s overall health. This would require further evolutionary studies. On the same note, it is theorised that robbing behaviour is an adaptation caused by human influence on honeybee colonies [18]. Adapting this mathematical model to an evolutionary framework can help address this question.

## Figures and Tables

**Table 1:** Parameter values and source references.

Parameter	Definition	Value	Ref.
*L*	maximum rate of egg laying	2000 eggs/day	[35]
*w*	number of hive bees for 50% egg survival	5000 bees	[35]
*b*	mass of food stored for 50% egg survival	500 g	[36]
*k*	increased food requirements for drone brood survival	variable	
α	maximum rate of recruitment	25%/day	[35]
$\frac{1}{\sigma }$	maximum fraction of colony that can be foraging	1/3	[35]
μ	natural death rate of foragers (summer)	0.14	[35]
$\mu_{D}$	natural death rate of drones	1/90	[39]
*c*	food gathered per day per forager	0.1 g/day/bee	[40]
$\gamma_{H}$	daily food requirement per adult hive bee	0.008 g/day/bee	[41]
$\gamma_{F}$	daily food requirement per adult forager	0.008 g/day/bee	[41]
$\gamma_{D}$	daily food requirement per adult drone	0.016 g/day/bee	calculated
$\gamma_{BH}$	daily food requirement per worker brood	0.016 g/day/bee	[42]
$\gamma_{BD}$	daily food requirement per drone brood	0.049 g/day/bee	[42]
*p*	proportion of worker eggs	0.95	[43]
β	rate of transmission of infection	$5\times 10^{-4}$	[23]
$d_{12}$	drift rate of foragers	0.1	[44]
${\hat{d}}_{12}$	drift rate of drones	$3d_{12}$	[15]

## Data Availability

Code available upon request.

## References

[1] Owen R. (2017). Role of human action in the spread of honey bee (Hymenoptera: Apidae) pathogens. J. Econ. Entomol..

[2] Coulon M., Dalmon A., Di Prisco G., Prado A., Arban F., Dubois E., Ribière-Chabert M., Alaux C., Thiéry R., Le Conte Y. (2020). Interactions between thiamethoxam and Deformed wing virus can drastically impair flight behavior of honey bees. Front. Microbiol..

[3] Flores J.M., Gil-Lebrero S., Gámiz V., Rodríguez M.I., Ortiz M.A., Quiles F.J. (2019). Effect of the climate change on honey bee colonies in a temperate Mediterranean zone assessed through remote hive weight monitoring system in conjunction with exhaustive colonies assessment. Sci. Total Environ..

[4] Colin T., Meikle W.G., Paten A.M., Barron A.B. (2019). Long-term dynamics of honey bee colonies following exposure to chemical stress. Sci. Total Environ..

[5] De Jong D. (1997). Mites: Varroa and other parasites of brood. Honey Bee Pests, Predators and Diseases.

[6] Wilfert L., Long G., Leggett H., Schmid-Hempel P., Butlin R., Martin S., Boots M. (2016). Deformed wing virus is a recent global epidemic in honeybees driven by Varroa mites. Science.

[7] Di Prisco G., Pennacchio F., Caprio E., Boncristiani H.F., Evans J.D., Chen Y. (2011). *Varroa destructor* is an effective vector of Israeli acute paralysis virus in the honeybee, *Apis mellifera*. J. Gen. Virol..

[8] Paxton R.J., Klee J., Korpela S., Fries I. (2007). *Nosema ceranae* has infected *Apis mellifera* in Europe since at least 1998 and may be more virulent than *Nosema apis*. Apidologie.

[9] Carreck N.L., Ball B.V., Martin S.J. (2010). The epidemiology of cloudy wing virus infections in honey bee colonies in the UK. J. Apic. Res..

[10] Ravoet J., De Smet L., Wenseleers T., De Graaf D.C. (2015). Genome sequence heterogeneity of Lake Sinai Virus found in honey bees and Orf1/RdRP-based polymorphisms in a single host. Virus Res..

[11] Spivak M., Gilliam M. (1998). Hygienic behaviour of honey bees and its application for control of brood diseases and Varroa: Part I. Hygienic behaviour and resistance to American foulbrood. Bee World.

[12] Spivak M. (1996). Honey bee hygienic behavior and defense against Varroa jacobsoni. Apidologie.

[13] Fefferman N.H., Traniello J.F., Rosengaus R.B., Calleri D.V. (2007). Disease prevention and resistance in social insects: Modeling the survival consequences of immunity, hygienic behavior, and colony organization. Behav. Ecol. Sociobiol..

[14] Moore A.J., Breed M.D., Moor M.J. (1987). The guard honey bee: Ontogeny and behavioural variability of workers performing a specialized task. Anim. Behav..

[15] Free J. (1958). The drifting of honey-bees. J. Agric. Sci..

[16] Goodwin R., Taylor M., Mcbrydie H., Cox H. (2006). Drift of Varroa destructor-infested worker honey bees to neighbouring colonies. J. Apic. Res. Bee World.

[17] Dynes T.L., Berry J.A., Delaplane K.S., Brosi B.J., de Roode J.C. (2019). Reduced density and visually complex apiaries reduce parasite load and promote honey production and overwintering survival in honey bees. PLoS ONE.

[18] Rittschof C.C., Nieh J.C. (2021). Honey robbing: Could human alterations to the environment change a rare foraging tactic into a maladaptive behavior?. Curr. Opin. Insect Sci..

[19] Peck D.T., Seeley T.D. (2019). Mite bombs or robber lures? The roles of drifting and robbing in Varroa destructor transmission from collapsing honey bee colonies to their neighbors. PLoS ONE.

[20] Graystock P., Goulson D., Hughes W.O. (2015). Parasites in bloom: Flowers aid dispersal and transmission of pollinator parasites within and between bee species. Proc. R. Soc. B Biol. Sci..

[21] Purkiss T., Lach L. (2019). Pathogen spillover from Apis mellifera to a stingless bee. Proc. R. Soc. B.

[22] Koch H., Brown M.J., Stevenson P.C. (2017). The role of disease in bee foraging ecology. Curr. Opin. Insect Sci..

[23] Betti M.I., Wahl L.M., Zamir M. (2014). Effects of Infection on Honey Bee Population Dynamics: A Model. PLoS ONE.

[24] Betti M.I., Wahl L.M., Zamir M. (2016). Reproduction Number and Asymptotic Stability for a Model with Continuous Age Structure: An Application to Honey Bee Dynamics. Bull. Math. Biol..

[25] Betti M., Wahl L., Zamir M. (2016). Age structure is critical to the population dynamics and survival of honeybee colonies. R. Soc. Open Sci..

[26] Muhammad N., Eberl H.J. (2020). Two routes of transmission for Nosema infections in a honeybee population model with polyethism and time-periodic parameters can lead to drastically different qualitative model behavior. Commun. Nonlinear Sci. Numer. Simul..

[27] Ratti V., Kevan P.G., Eberl H.J. (2013). A mathematical model for population dynamics in honeybee colonies infested with *Varroa destructor* and the Acute Bee Paralysis Virus. Can. Appl. Math. Q..

[28] Eberl H.J., Frederick M.R., Kevan P.G. (2010). Importance of brood maintenance terms in simple models of the honeybee—*Varroa destructor*—Acute Bee Paralysis Virus complex. Electron. J. Differ. Equ..

[29] Petric A., Guzman-Novoa E., Eberl H.J. (2017). A mathematical model for the interplay of Nosema infection and forager losses in honey bee colonies. J. Biol. Dyn..

[30] Muhammad N., Eberl H.J. (2017). A simple model of between-hive transmission of Nosemosis. International Conference on Applied Mathematics, Modeling and Computational Science.

[31] Bartlett L.J., Rozins C., Brosi B.J., Delaplane K.S., de Roode J.C., White A., Wilfert L., Boots M. (2019). Industrial bees: The impact of apicultural intensification on local disease prevalence. J. Appl. Ecol..

[32] Le Conte Y., Ellis M., Ritter W. (2010). Varroa mites and honey bee health: Can Varroa explain part of the colony losses?. Apidologie.

[33] Betti M., LeClair J., Wahl L.M., Zamir M. (2017). Bee++: An Object-Oriented, Agent-Based Simulator for Honey Bee Colonies. Insects.

[34] Fries I. (1993). *Nosema apis*—A parasite in the honey bee colony. Bee World.

[35] Khoury D.S., Myerscough M.R., Barron A.B. (2011). A Quantitative Model of Honey Bee Colony Population Dynamics. PLoS ONE.

[36] Khoury D.S., Barron A.B., Myerscough M.R. (2013). Modelling Food and Population Dynamics in Honey Bee Colonies. PLoS ONE.

[37] Heidinger I.M.M., Meixner M.D., Berg S., Büchler R. (2014). Observation of the mating behavior of honey bee (*Apis mellifera* L.) queens using radio-frequency identification (RFID): Factors influencing the duration and frequency of nuptial flights. Insects.

[38] Seeley T., Mikheyev A. (2003). Reproductive decisions by honey bee colonies: Tuning investment in male production in relation to success in energy acquisition. Insectes Sociaux.

[39] Fukuda H., Ohtani T. (1977). Survival and life span of drone honeybees. Popul. Ecol..

[40] Russell S., Barron A.B., Harris D. (2013). Dynamic modelling of honey bee (*Apis mellifera*) colony growth and failure. Ecol. Model..

[41] Brodschneider R., Crailsheim K. (2010). Nutrition and health in honey bees. Apidologie.

[42] Hrassnigg N., Crailsheim K. (2005). Differences in drone and worker physiology in honeybees (*Apis mellifera*). Apidologie.

[43] Jay S. (1974). Seasonal development of honeybee colonies started from package bees. J. Apic. Res..

[44] Neumann P., Moritz R.F., Mautz D. (2000). Colony evaluation is not affected by drifting of drone and worker honeybees (*Apis mellifera* L.) at a performance testing apiary. Apidologie.

[45] Free J., Racey P. (1968). The effect of the size of honeybee colonies on food consumption, brood rearing and the longevity of the bees during winter. Entomol. Exp. Appl..

[46] Downs S.G., Ratnieks F.L. (2000). Adaptive shifts in honey bee (*Apis mellifera* L.) guarding behavior support predictions of the acceptance threshold model. Behav. Ecol..

[47] Pfeiffer K., Crailsheim K. (1998). Drifting of honeybees. Insectes Sociaux.

[48] Wolf S., Nicholls E., Reynolds A.M., Wells P., Lim K.S., Paxton R.J., Osborne J.L. (2016). Optimal search patterns in honeybee orientation flights are robust against emerging infectious diseases. Sci. Rep..

[49] Milbrath M.O., van Tran T., Huang W.F., Solter L.F., Tarpy D.R., Lawrence F., Huang Z.Y. (2015). Comparative virulence and competition between Nosema apis and Nosema ceranae in honey bees (*Apis mellifera*). J. Invertebr. Pathol..

[50] Mattila H., Otis G. (2006). Effects of pollen availability and Nosema infection during the spring on division of labor and survival of worker honey bees (Hymenoptera: Apidae). Environ. Entomol..

[51] Botías C., Martín-Hernández R., Días J., García-Palencia P., Matabuena M., Juarranz Á., Barrios L., Meana A., Nanetti A., Higes M. (2012). The effect of induced queen replacement on Nosema spp. infection in honey bee (*Apis mellifera* iberiensis) colonies. Environ. Microbiol..

[52] Liess M., Foit K., Knillmann S., Schäfer R.B., Liess H.D. (2016). Predicting the synergy of multiple stress effects. Sci. Rep..

[53] Comper J.R., Eberl H.J. (2020). Mathematical modelling of population and food storage dynamics in a honey bee colony infected with Nosema ceranae. Heliyon.

[54] Geffre A.C., Gernat T., Harwood G.P., Jones B.M., Gysi D.M., Hamilton A.R., Bonning B.C., Toth A.L., Robinson G.E., Dolezal A.G. (2020). Honey bee virus causes context-dependent changes in host social behavior. Proc. Natl. Acad. Sci. USA.

[55] Bromenshenk J.J., DeGrandi-Hoffman G. (1993). PC BEEPOP: A Microcomputer Model of the Population Dynamics and Economics of Honey Bee Colonies. Am. Entomol..

[56] Fürst M., McMahon D.P., Osborne J., Paxton R., Brown M. (2014). Disease associations between honeybees and bumblebees as a threat to wild pollinators. Nature.

[57] Manley R., Boots M., Wilfert L. (2015). Emerging viral disease risk to pollinating insects: Ecological, evolutionary and anthropogenic factors. J. Appl. Ecol..

[58] Dalmon A., Diévart V., Thomasson M., Fouque R., Vaissière B.E., Guilbaud L., Le Conte Y., Henry M. (2021). Possible spillover of pathogens between bee communities foraging on the same floral resource. Insects.

